# Revisiting the paradigm of silica pathogenicity with synthetic quartz crystals: the role of crystallinity and surface disorder

**DOI:** 10.1186/s12989-016-0136-6

**Published:** 2016-06-10

**Authors:** Francesco Turci, Cristina Pavan, Riccardo Leinardi, Maura Tomatis, Linda Pastero, David Garry, Sergio Anguissola, Dominique Lison, Bice Fubini

**Affiliations:** 1 Department of Chemistry, University of Torino, Via P. Giuria 7, Turin, 10125 Italy; 2 “G. Scansetti” Interdepartmental Center for Studies on Asbestos and Other Toxic Particulates, University of Torino, Via P. Giuria 7, Turin, 10125 Italy; 3 Department of Earth Sciences, University of Torino, Via V. Caluso 35, Turin, 10125 Italy; 4 Centre for BioNano Interactions, School of Chemistry and Chemical Biology, University College Dublin, Dublin, Belfield Ireland; 5 Louvain centre for Toxicology and Applied Pharmacology (LTAP), Université catholique de Louvain, Avenue E. Mounier 52 – bte B1.52.12, Brussels, 1200 Belgium

**Keywords:** Quartz, Silica, Toxicity, Silanols, Crystallinity, Membranolysis, Conchoidal fracture, Surface charge, Radicals

## Abstract

**Background:**

Exposure to some - but not all - quartz particles is associated to silicosis, lung cancer and autoimmune diseases. What imparts pathogenicity to any single quartz source is however still unclear. Crystallinity and various surface features are implied in toxicity. Quartz dusts used so far in particle toxicology have been obtained by grinding rocks containing natural quartz, a process which affects crystallinity and yields dusts with variable surface states. To clarify the role of crystallinity in quartz pathogenicity we have grown intact quartz crystals in respirable size.

**Methods:**

Quartz crystals were grown and compared with a fractured specimen obtained by grinding the largest synthetic crystals and a mineral quartz (positive control). The key physico-chemical features relevant to particle toxicity - particle size distribution, micromorphology, crystallinity, surface charge, cell-free oxidative potential - were evaluated. Membranolysis was assessed on biological and artificial membranes. Endpoints of cellular stress were evaluated on RAW 264.7 murine macrophages by High Content Analysis after ascertaining cellular uptake by bio-TEM imaging of quartz-exposed cells.

**Results:**

Quartz crystals were grown in the submicron (n-Qz-syn) or micron (μ-Qz-syn) range by modulating the synthetic procedure. Independently from size as-grown quartz crystals with regular intact faces did not elicit cellular toxicity and lysosomal stress on RAW 264.7 macrophages, and were non-membranolytic on liposome and red blood cells. When fractured, synthetic quartz (μ-Qz-syn-f) attained particle morphology and size close to the mineral quartz dust (Qz-f, positive control) and similarly induced cellular toxicity and membranolysis. Fracturing imparted a higher heterogeneity of silanol acidic sites and radical species at the quartz surface.

**Conclusions:**

Our data support the hypothesis that the biological activity of quartz dust is not due to crystallinity but to crystal fragmentation, when conchoidal fractures are formed. Besides radical generation, fracturing upsets the expected long-range order of non-radical surface moieties - silanols, silanolates, siloxanes - which disrupt membranes and induce cellular toxicity, both outcomes associated to the inflammatory response to quartz.

**Electronic supplementary material:**

The online version of this article (doi:10.1186/s12989-016-0136-6) contains supplementary material, which is available to authorized users.

## Background

Excessive exposure to crystalline silica dusts is associated with silicosis, lung cancer and autoimmune diseases, and the crystalline structure has been traditionally considered as a key determinant of silica pathogenicity. However, not all crystalline silica dusts are pathogenic [1, 2]. A preamble in IARC monograph volume 68 recalls that carcinogenicity of crystalline silica dust does not apply to all occupational exposures examined [1].

Several amorphous silica particles have the potential to cause adverse cellular effects associated with the development of silica-related pathologies [3–14]. Vitreous silica particles obtained by grinding a pure silica glass exhibited all the physico-chemical features of quartz dusts except crystallinity [15], and the same biological reactivity as quartz in vitro [3]. Some cases of silicosis and lung cancer were also reported among workers exposed to this type of amorphous silica [16, 17]. Thus, the traditional paradigm confining adverse effects exclusively to crystalline silica comes under discussion. Furthermore, a large amount of studies on amorphous nanosilicas have shown remarkable in vitro toxic responses [4–9], and in vivo, transient, adverse pulmonary effects [10–14]. Even for amorphous nanosilicas, toxic responses markedly varied from one type to the other [11].

This variability of silica hazard stems from both the chemical nature of the dust and the multiple interactions of silica particles with biomolecules and cells within the respiratory system, each interaction being possibly modulated by the different physico-chemical features of the particles [2, 18]. In spite of a large body of studies, the role of each physico-chemical property in triggering a specific biological event remains unclear, mainly because of (1) the intrinsic variability of the mineral sources of quartz investigated, and (2) the variability induced during industrial processing which largely alters many key surface features [19–23], thus making the toxicity of each silica dust quite unpredictable.

All silicas share a [SiO_4_]^4−^ tetrahedron as basic unit and may differ by their spatial arrangement. As a consequence, several crystalline polymorphs as well as many kinds of amorphous specimens exist [24], differing in surface chemical functionalities as well as in biological reactivity and pathogenicity [18, 25]. During mechanical fracturing, silicon-oxygen bonds, being covalent and slightly polar, yields both surface radicals (dangling bonds) and charges. Upon surface reconstruction with ambient water vapour, a complex array of silanol families (Si-OH) and siloxane bridges (Si-O-Si) form rings of different sizes, whose kinetics of formation and stability are strongly dependent upon the origin, mechanical, chemical, and thermal history of the particles.

To revisit the role of crystallinity in the pathogenicity of silica particles, we have grown and tested two preparations of quartz crystals with intact and unaltered surfaces. Both crystals were mostly in the respirable size, ranging from submicrometric (n-Qz-syn) to micrometric (μ-Qz-syn). A portion of the micrometric quartz was then mechanically fractured (μ-Qz-syn-f) to induce structural surface alterations, including radicals and new silanol distributions on freshly formed surfaces. A well-characterized pure quartz dust (Qz-f), obtained by grinding large quartz crystals from Madagascar and previously used in toxicity tests [3], was included in each experiment as positive control. The four quartz samples were fully characterized for morphology and crystallinity (SEM, HR-TEM, XRD), particle size distribution (differential centrifugal sedimentation, DCS), surface area (BET), heterogeneity of surface acidic silanols (electrophoretic light scattering, ELS), and radical-mediated surface reactivity (spin trapping/EPR spectroscopy). We compared as-grown crystals vs fractured ones in a series of cellular and acellular assays relevant for the pathogenicity of silica particles, including i) membranolytic activity towards biological (red blood cells, RBCs) and artificial (liposomes) membranes, ii) cell viability and stress-related endpoints (high content analysis, HCA) in murine macrophages, and iii) particle uptake/internalization (bio-TEM).

## Results

### Synthetic quartz was grown in different sizes with intact surfaces, and then fractured to obtain features similar to ground mineral quartz dust

**Fig. 1:**
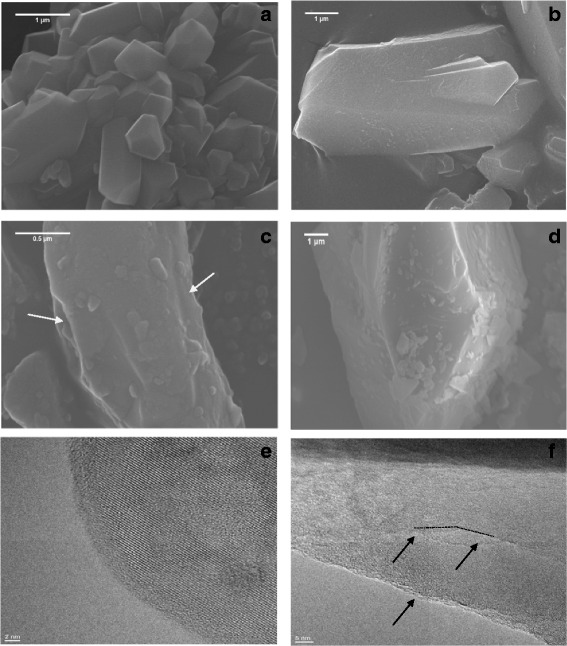
FE-SEM imaging of n-Qz-syn, μ-Qz-syn, μ-Qz-syn-f, and Qz-f (**a**, **b**, **c**, and **d**, respectively) and HR-TEM imaging of as-grown (n-Qz-syn) and fractured (μ-Qz-syn-f) samples (**e** and **f**, respectively). Flat, well-formed, smooth surfaces characterized as-grown quartz (**a** and **b**), while conchoidal fractures (arrow) were visible on ground particles (**c** and **d**). Crystal structure was preserved up to the very last atomic boundary of the synthetic crystal (**e**), while fracturing induced partial disorganization of crystal lattice (absence of diffraction fringes) and loss of long-range ordered crystalline planes (**f**, arrows)

Two synthetic quartz crystals with controlled properties were produced with a novel approach described in a parallel paper devoted to the mineralogical details of the crystal synthesis [26]. The technique adopted allowed modulation of crystal size, while keeping the lattice distortion to a minimum, close to the ideal structure of quartz. Size and surface area of as-grown and fractured quartz crystals employed in the study are reported in Table 1. The particle size of the two as-grown crystals was largely different and surface area varied with particle size accordingly. The scanning electron microscopy (SEM) images of the two types of synthetic crystals are reported in Fig. 1a and b. The n-Qz-syn exhibited regular shape and very fine particle size (from 100 to 400 nm). Hexagonal-shaped sub-micrometric crystals were often observed for this sample. Larger crystals of μ-Qz-syn had a less regular morphology and a higher size range (up to ca. 2500 nm). Both crystals exhibited smooth and regular surfaces [26]. A representative HR-TEM image of as-grown synthetic quartz crystals is reported in Fig. 1e. Single ordered lattice layers (diffraction fringes) were observed to be extending over the whole crystal particle, including the regions close to the surfaces.

**Table 1:** Size and specific surface area of the studied quartz crystals

Crystal type	Acronym	Particle diameter 10th percentile (nm)^a^	Particle diameter 50th percentile (nm)^a^	Particle diameter 90th percentile (nm)^a^	Surface area (m^2^/g)^b^
Synthetic as-grown crystals in submicron size	n-Qz-syn	174	276	408	6.2
Synthetic as-grown crystals in micron size	μ-Qz-syn	405	904	2520	0.3
Fractured micron size synthetic crystals	μ-Qz-syn-f	409	914	1960	9.5
Mineral quartz dust (positive control)	Qz-f	285	704	1290	6.1

^a^Measured by Differential Centrifugal Sedimentation (DCS), see Additional file 1: Figure S1

^b^Measured by Kr-BET method

Mechanical fracturing of μ-Qz-syn crystals caused the formation of conchoidal fractures [27, 28] and sub-micrometric particles electrostatically stacked on larger crystals, clearly visible in the SEM image of μ-Qz-syn-f (Fig. 1c) and responsible for the significant increase in surface area (from 0.3 to 9.5 m^2^/g). Similar fractures and surface area were evidenced for ground mineral quartz dust, Qz-f (Fig. 1d and Table 1). HR-TEM performed on fractured synthetic quartz (μ-Qz-syn-f) highlighted a large amount of defects and disordered structures introduced into the crystal lattice, with consequent loss of long-range crystal planes (Fig. 1f, arrows).

### Contrary to fractured quartz, as-grown crystals do not reduce cell viability or induce cellular stress in RAW 264.7 macrophages

**Fig. 2:**
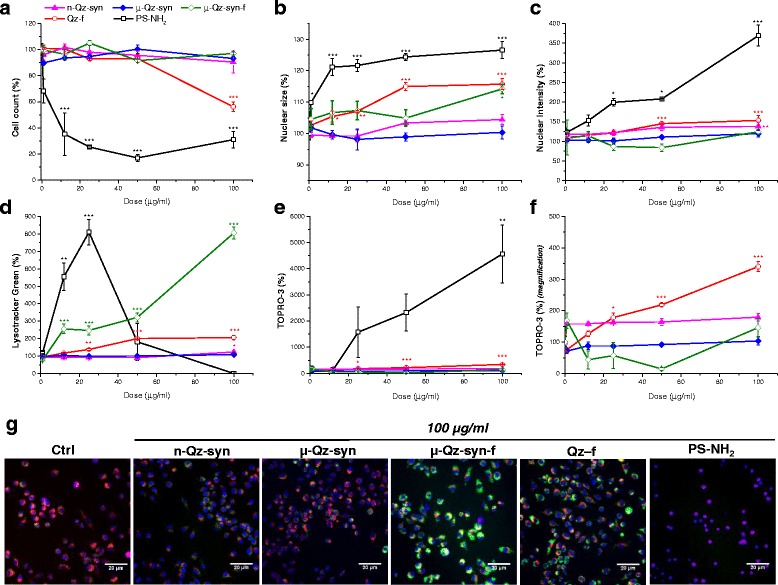
Cell toxicity in RAW 264.7 macrophages exposed to as-grown (n-Qz-syn and μ-Qz-syn) or fractured (μ-Qz-syn-f and Qz-f) quartz crystals. RAW 264.7 murine macrophages were exposed for 24 h to medium (Ctrl) or increasing concentrations of n-Qz-syn, μ-Qz-syn, μ-Qz-syn-f, Qz-f, and PS-NH_2_ beads (cytotoxic control). **a** Cell count (number of Hoechst stained nuclei), **b** nuclear size (average object area of Hoechst), **c** nuclear intensity (Hoechst intensity), **d** lysosomal acidification (Lysotracker Green intensity), and **e** plasma membrane integrity (TOPRO-3 intensity) were measured. A magnification from 0 to 500 % of TOPRO-3 intensity plot is given in panel **f**. As-grown quartz crystals (n-Qz-syn and μ-Qz-syn) were inactive for all the cytotoxicity parameters investigated, while fractured quartz (μ-Qz-syn-f and Qz-f) induced significant cell stress. Data are reported as mean percentages of the control ± SEM in a representative experiment performed in triplicate. *p < 0.05, **p < 0.01 and ***p < 0.001 vs control not exposed to quartz. Representative images captured by epifluorescence microscopy on RAW 264.7 macrophages exposed to Ctrl or quartz samples at 100 μg/ml (**g**). Blue fluorescence is indicative of nuclear staining, green fluorescence of acidic compartment staining, red fluorescence of mitochondrial membrane potential, and violet fluorescence of cell membrane permeability. Scale bars = 20 μm

Cytotoxic profiles of the as-grown quartz crystals (n-Qz-syn, μ-Qz-syn) were examined by means of High Content Analysis (HCA) [5, 29, 30] and compared with those of the fractured crystals (μ-Qz-syn-f) and the ground mineral quartz dust (Qz-f). RAW 264.7 macrophages were exposed for 24 h over a range of particle concentrations (1, 12, 25, 50 and 100 μg/ml). After exposure (Fig. 2) as-grown quartz crystals (n-Qz-syn and μ-Qz-syn) did not cause any alteration of the five cytotoxicity parameters assessed. In contrast, fractured synthetic quartz (μ-Qz-syn-f) elicited an increase of cell nuclear size at the highest dose (100 μg/ml) and a remarkable dose-dependent increase of lysosomal acidification. Lysosomal acidification and increase of nuclear size were also recorded in cells incubated with mineral quartz dust (Qz-f), which also reduced cell count and increased cell membrane permeability.

These quantitative data are supported by imaging (Fig. 2g) of RAW 264.7 macrophages exposed to medium (Ctrl) or to 100 μg/ml of n-Qz-syn, μ-Qz-syn, μ-Qz-syn-f, Qz-f, and PS-NH_2_ (cytotoxic control). Control cells (Ctrl) showed blue and red fluorescence reflecting nuclear staining and intact mitochondria, respectively. n-Qz-syn and μ-Qz-syn were similar to Ctrl. μ-Qz-syn-f and Qz-f induced an increase of the green staining, due to lysosomal acidification. A violet fluorescence staining due to plasma membrane permeabilization was observed for the cytotoxic control.

Bio-TEM images of RAW 264.7 macrophages incubated with quartz particles for 24 h confirmed that all samples were internalized (Additional file 1: Figure S2).

### Contrary to fractured quartz, as-grown quartz crystals show a low membranolytic activity towards RBCs and small liposomes

**Fig. 3:**
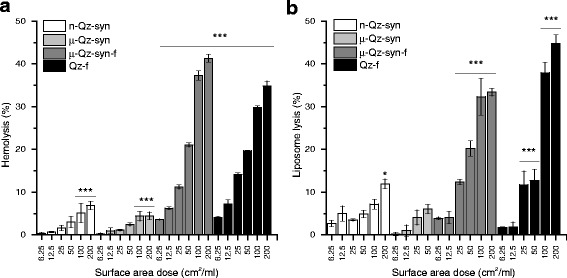
Hemolysis (**a**) and liposome rupture (**b**) induced by as-grown (n-Qz-syn, μ-Qz-syn) and fractured (μ-Qz-syn-f and Qz-f) quartz crystals. Quartz was incubated at increasing concentrations with purified human red blood cells (RBCs) or phosphatidylcholine vesicles. Values are mean ± SD from at least three independent experiments. *p < 0.05, **p < 0.01 and ***p < 0.001 vs control not exposed to particles. A modest lytic effect towards RBCs and small lipid vesicles (<300 nm) was observed upon incubation with as-grown quartz crystals, while a remarkable dose-dependent lysis, up to ca. 45 %, was induced by fractured quartz

As-grown quartz crystals (n-Qz-syn and μ-Qz-syn) incubated with purified human red blood cells (RBCs) induced only a modest hemolytic activity (Fig. 3a). On the contrary, fractured synthetic quartz (μ-Qz-syn-f) showed a strong dose-dependent hemolytic activity. The interaction of quartz with artificial, small phosphatidylcholine liposomes (hydrodynamic diameter peaks at ca. 50 and 280 nm, measured with DLS – Additional file 1: Figure S3) was assessed by a fluorescent probe leakage assay. The membranolytic activity towards liposomes (Fig. 3b) confirmed the low and the strong activity of as-grown quartz crystals and fractured ones, respectively. Consistently, the ground mineral quartz dust (Qz-f) showed a high lytic activity in both assays.

### Fractured particles exhibit a less homogeneous distribution of surface silanol acidity than as-grown quartz crystals

**Fig. 4:**
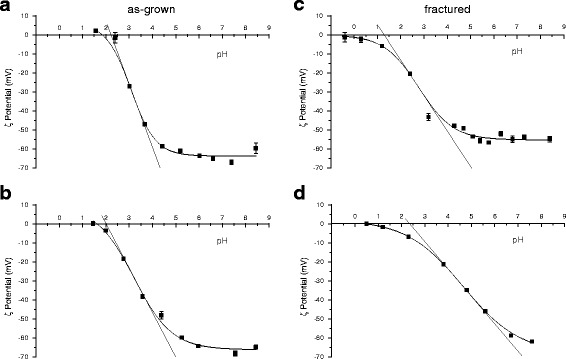
Z plot (ζ potential vs pH) of as-grown n-Qz-syn (**a**) and μ-Qz-syn (**b**), and fractured μ-Qz-syn-f (**c**) and Qz-f (**d**) quartz crystals. Experimental points acquired with at least three measurements are fitted with a non-linear curve to help the reader’s eye. The slope of the tangent line at the inflection point is a convenient estimate of the heterogeneity of surface silanol acidity (see Additional file 1: Table S1). The steeper the slope of the tangent line the more homogeneous the acidic nature of the silanols at the surface

The chemical homogeneity of the surface acidic functionalities (i.e. silanols) of the four samples was investigated by evaluating the variations of their ζ potential in aqueous medium as a function of pH. Surface charge is a function of the equilibrium between the protonated and dissociated silanols [31]. Silanols are Brønsted acidic sites and the equilibrium condition can be described with a “titration curve” (ζ plot) performed by acidifying the medium while the surface net charge of the suspended quartz is measured by ELS (Fig. 4) [32]. In alkaline conditions the vast majority of silanols on all quartz crystals were dissociated, resulting in a markedly negative surface zeta potential (ca. −65 mV). By decreasing the pH, the negative surface charge was progressively reduced and approached the point of zero charge (PZC), asymptotically. All curves exhibited a sigmoid pattern, but the slope of the two as-grown crystals (a, b) was much steeper than that of the fractured ones, both synthetic (c) and mineral (d) (see Additional file 1: Table S1), reflecting a greater heterogeneity of silanols at the surface of fractured particles.

### Surface radicals are generated after grinding synthetic as-grown quartz crystals

**Fig. 5:**
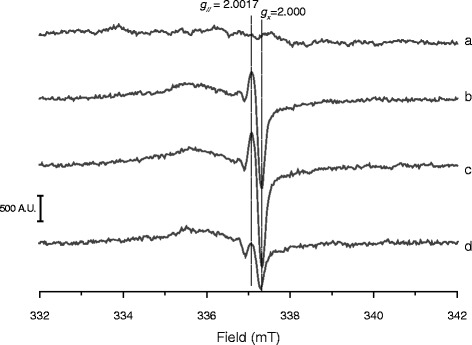
Solid state EPR spectra of μ-Qz-syn crystal recorded at −196 ° C: as-grown (**a**), as milled (**b**), 3 days after milling (**c**), and 1 month after milling (**d**). The spectra were recorded with: sweep width 100 G, receiver gain 1 × 10^4^, microwave power 0.1 mW (a very low potency at which the silyl radical is visible and not saturated, while all the other silico-oxygen radical features are hardly visible), scan time 80 s. Mechanical fracturing of as-grown μ-Qz-syn introduced radical defects (**b**), absent on the pristine quartz crystal (**a**). Two components (g_//_and g_x_) of the signal relative to silyl radicals were detected. Silyl radicals were stable for three (**c**) and decreased 30 days after milling (**d**)

The surface radicals of quartz particles, generated by cleavage of the Si-O-Si bonds during grinding and subsequent reaction with atmospheric water and O_2_ molecules, have been extensively described [15, 33, 34]. The presence of surface radicals in quartz was often related to the potential to generate free radicals [35–37] hence promoting pathogenicity [38, 39]. Figure 5 reports the solid state EPR (SS-EPR) spectra of as-grown μ-Qz-syn (a) and the same crystal mechanically fractured (μ-Qz-syn-f) after 0 (b), 3 (c), and 30 days (d). Remarkably, as-grown μ-Qz-syn crystals did not show any radical specie. Right after mechanical fracturing, SS-EPR spectrum was characterized by an intense signal at *g* values (defined by the resonance condition *g* = *hν*_0_/*μ*_B_ *B*, in which *B* is the resonant field and *ν*_0_ the applied microwave frequency) of g_//_= 2.000 and g_x_ = 2.0017 (μ-Qz-syn-f, spectrum b), corresponding to the parallel and *x*-axis component of the silyl radical (Si^•^), respectively [33]. The radical signal was stable up to three days (spectrum c). Other silicon and oxygen based radicals (for instance, SiO^•^, SiO_2_^•^, SiO_3_^•^, O_2_^•^ˉ) could also be detected after fracturing as revealed by the complex convoluted signal at much lower magnetic field [40]. After a longer time (30 days, spectrum d) silyl radicals decreased in intensity, probably due to surface reconstruction [37]. Notably, all spectra did not exhibit paramagnetic centres arising from metal ions occluded, confirming the high purity of the crystals obtained.

### Fractured, but not as-grown, quartz crystals generate carbon-centred radicals

**Fig. 6:**
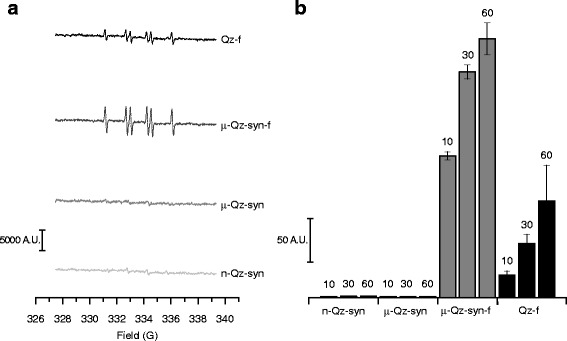
Carbon-centred free radicals generated in an aqueous suspension containing sodium formate after incubation with quartz particles for 10, 30 and 60 min. Carbon-centred radicals result from homolytic cleavage of a C-H bond in the formate ion. Representative spectra in panel (**a**) were collected after 60 min of incubation. The signal intensity is proportional to the amount of radicals generated. The kinetics of carboxyl radical formation (**b**) was calculated by double-integration of the peak areas of collected spectra and expressed in arbitrary units. Experiments were performed in duplicate and reported as means ± SD. While as-grown quartz crystals (μ-Qz-syn, n-Qz-syn) showed no radical reactivity, fracturing (μ-Qz-syn-f) induced a strong, sustained reactivity

All quartz samples were tested for their potential to generate carbon-centred radicals after 10, 30 and 60 min of incubation with formate ion. The homolytic cleavage of the hydrogen-carbon bond in formate was followed by spin trapping technique and quantified with EPR spectroscopy, as carried out in several previous studies with quartz dusts [20, 41–43]. Representative EPR/spin trapping spectra of the [DMPO–COO]^• –^ adduct obtained in the presence of sodium formate at 60 min and double-integration of the reaction kinetics are reported in Fig. 6a and b, respectively. No radical was observed with as-grown quartz crystals (μ-Qz-syn, n-Qz-syn), whereas freshly fractured quartz (μ-Qz-syn-f) had a strong ability to generate carbon-centred free radicals in solution, even more than ground mineral quartz (Qz-f). The strong reactivity of fractured crystals was sustained over time, up to 60 min.

## Discussion

This study reveals, for the first time, that disorder in the crystal lattice drives the biological reactivity of quartz, leading to a new paradigm of toxicity. Crystallinity, so far held responsible for the adverse effects of silica [1, 2], does not appear related *per se* to toxicity because quartz crystals grown with intact faces were inert in several cellular and cell-free tests relevant for the pathogenicity of inhaled silica particles.

We newly synthesized model quartz crystals to overcome the variability of mineral quartz dusts, usually obtained by grinding, that have been used in previous toxicological and mechanistic investigations. The synthetic procedure adopted in this work yielded quartz crystals with intact surfaces, in submicron or micron size (depending on the synthetic environment) in the respirable dimension. Usually, in silica or silica-related specimens, the near-to-surface regions often appear amorphous, even in samples with good crystallinity at the core [15, 44–46]. In the present case, the surface of as-grown quartz crystals was a well-formed, ordered crystal face. These as-grown quartz crystals were readily internalized by macrophages, but did not elicit evident cytotoxic or cell-stress responses, in particular lysosomal acidification, in HCA tests. Furthermore, they did not exhibit membranolytic activity in biological or artificial membrane models. The large differentiation in size of the two as-grown crystals led us to exclude that particle size alone could be a key parameter in eliciting the biological reactivity of quartz. This is consistent with previous findings, showing that the in vivo pulmonary toxicity of quartz particles correlates better with surface activity than particle size and surface area [47].

Upon fracturing, conchoidal surface fractures, characterized by a less ordered lattice structure [27, 28] and a morphology typical of ground mineral quartz, were observed. Under these experimental conditions, the dramatic increase of specific surface area (from 0.3 to 9 m^2^/g, for μ-Qz-syn and μ-Qz-syn-f respectively) did not parallel the lowering in particle size. This confirms that the main modification which is induced by fracturing occurs at the surface level, determining the formation of highly irregular particles with an increased specific surface area. Stable surface radicals and the ability to form carbon-centred radicals, which have an essential role in promoting the complex mechanisms of quartz pathogenicity [2, 39, 48–50], were measured. Fractured quartz crystals induced, like ground mineral quartz, strong lysosomal stress to RAW 264.7 cells, as well as RBC and liposome lytic activity.

The long-range order of surface functionalities present on as-grown crystal faces was partially lost upon grinding. The progressive surface reconstruction can leave hydrophilic and hydrophobic patches and disordered silanols arrays similar to the surface of some amorphous silicas [24, 51, 52] also reported as highly hemolytic [5, 53]. Fractured quartz exhibited a higher heterogeneity of the surface acidic sites (silanols) compared to the as-grown crystals (ζ plot). The steeper slopes measured for as-grown quartz indicate that silanols on intact surfaces have mostly the same acidity, while the lower slopes of fractured quartz indicate a greater silanol heterogeneity, due to a less spatially-ordered state of polar moieties, including silanols. This finding is also supported by the structural disorder of the crystalline lattice observed with HR-TEM. These irregular surfaces were here shown to strongly interact with membranes inducing lysis. Congruently, previous studies associated the hemolytic activity to some peculiar spatial arrangements of silanols and siloxanes [51, 53, 54].

We also evaluated the interaction between nano-sized lipid vesicles and quartz crystals through a liposome leakage assay [55]. The results of liposome lysis were similar to those obtained with RBC, even if the sizes of the membrane models largely differed (<300 nm liposome, ca. 7 μm RBC). The small dimension of liposomes allowed to probe nanometric patches on the quartz surface and to rule out the hypothesis that micrometric features on the quartz crystals (such as spikes and sharp edges) may have a role in determining the interaction between quartz and membranes.

Together, the data are consistent with the notion that most of the biological reactivity of quartz dusts is originated via fragmentation, when cell membranes and tissues interact with conchoidal fractures and not with intact as-grown crystal faces. Conchoidal fracture generates surface radicals and, cutting across several crystal planes, a disordered array of silanols, siloxanes and rings. Surface modifications of quartz dusts which reduce silanol heterogeneity, e.g. hydration [53], surface coverage by polymers [56, 57], reaction of silanols with metal ions [56, 58–61] were shown to reduce or blunt silica toxicity, likely by avoiding the phagolysosomal rupture within macrophages and inflammatory responses [19, 62–65].

## Conclusion

As-grown synthetic quartz crystals did not show biological reactivity in a series of toxicologically relevant tests, indicating that crystallinity *per se* does not appear as a key determinant of the pathogenic activity of silica particles. A regular distribution of the silanols at the particle surface was shown to occur in synthetic as-grown quartz crystals. Fracturing led to a disorganization of these surface moieties, causing a loss of the long-range spatial order and probably creating reactive surface silanol patches. Biological reactivity, and possibly toxicity, appears related with disordered surface functionalities following crystal fracture.

## Methods

### Chemical reagents

Dulbecco’s modified Eagle medium (DMEM) glutamax, fetal bovine serum (FBS) and penicillin-streptomycin were purchased from Life Technologies. NaCl 0.9 % was obtained from B. Braun Medical (Diegem, Belgium), Triton X-100 from Flucka (Buchs, Switzerland). The fluorescent dyes, Hoechst 33342, TMRM, Lysotracker green and TOPRO-3 were purchased from Life Technologies. All the reagents used for liposome synthesis (methanol, chloroform, L-α-phosphatidylcholine, and calcein) were purchased from Sigma-Aldrich (Milan, Italy). Ultrapure Milli-Q water (Millipore) was used throughout.

### Synthetic and mineral quartz

Four powdered quartz samples of synthetic or mineral origin have been here investigated. As-grown quartz crystals (n-Qz-syn and μ-Qz-syn) were obtained by hydrothermal synthesis following a procedure well described in [26]. Briefly, a 25 % w/w sodium metasilicate pentahydrate solution (Na-MTS, Sigma-Aldrich) was polymerized using two mineral acids, i) HNO_3_ (for n-Qz-syn), and ii) H_2_CO_3_ obtained by bubbling CO_2_ into the Na-MTS solution until gel formation (for μ-Qz-syn). The gel was stabilized at pH ≈ 8. Growth runs were performed in PFTE liner sealed into steel autoclaves at 200 ° C and autogenic pressure for 168 h. Fractured μ-Qz-syn-f was obtained by milling 75 mg of μ-Qz-syn in a ball mill (Retsch MM200) in agate jars (27 Hz, with 2 agate spheres) for 30 min. One pure quartz dust (Qz-f) of mineral origin was prepared by grinding a very pure quartz crystal from Madagascar in a planetary ball mill (Retsch S100, GmbH, Haan, Germany) for 3 h (70 rpm) and then in the ball mill for 9 h (27 Hz).

### Differential centrifugal sedimentation (DCS)

Particle size distribution was determined by differential centrifugal sedimentation using a CPS Disc Centrifuge DC24000 (CPS Instruments Europe) operating at 14000× g with a 6–24 % sucrose gradient in phosphate buffer solution (PBS, pH 7.4). Further details on the technique adopted are reported in [66]. Each experiment has been repeated at least twice.

### Surface area determination

The surface area of the quartz particles was measured using the BET method based on Kr or N_2_ adsorption, as appropriate. Quartz samples have been degassed for hours prior to analysis, which was carried out at −196 ° C (ASAP 2020 Micromeritics, Norcross, USA).

### Field emission scanning electron microscopy (FE-SEM)

Sample morphology was investigated by Scanning Electron Microscopy (SEM) using a Zeiss Evo50 and a Hitachi S-4300 Field Emission SEM. Quartz samples were sonicated for 30 min in ultrapure water, dropped off on conductive stubs, and coated with gold in order to prevent the electron beam from charging the sample. The operating conditions were: EHT 15 to 25 kV, WD 1 to 6 mm, probe current 100 pA.

### High resolution transmission electron microscopy (HR-TEM)

To inspect the crystal lattice and the defectiveness of the quartz samples, high resolution transmission electron microscopy was carried out with a JEOL 3010-UHR, equipped with a LaB_6_ filament operating at 300 kV, beam current 114 μA and with a 2 k × 2 k pixels Gatan US1000 CCD camera. Quartz samples were dispersed in ultrapure water, sonicated for 20 min and a droplet was deposited on lacey carbon Cu grids.

### Cell toxicity assessment by high content analysis (HCA) on RAW 264.7 murine macrophages

High Content Analysis (HCA) for toxicological investigation [5, 30, 67] was used to screen quartz samples for cell viability and stress-related responses. The protocol refers to [29]. Briefly, RAW 264.7 murine macrophages (purchased from ECACC) were grown to pre-confluence at 37 ° C in a 5 % CO_2_-supplemented atmosphere in DMEM glutamax supplemented with 10 % FBS, 1 % penicillin (100 U/ml) and streptomycin (100 μg/ml). 5 × 10^3^ cells/well were seeded in a 96-well tissue culture plate and allowed to adhere in 100 μl of DMEM glutamax supplemented with 10 % FBS for 16–24 h (37 ° C, 5 % CO_2_) before particle incubation. Stock suspensions were prepared by suspending 2 mg/ml of quartz particles in PBS and sonicated for 10 min with an ultrasonic bath (Branson, Bransonic 1510; 80 W). Particle dispersions were prepared by diluting sample stock suspensions in the culture media to 3 × the final concentration required; then, 50 μl of particle suspensions were distributed in cell culture plates, obtaining the final concentrations of 1, 12, 25, 50 and 100 μg/ml. A negative control (only culture medium) and a cytotoxic control (NH_2_-conjugated polystyrene beads, PS-NH_2_) [29] were also included. Cells were incubated with quartz particles for 24 h (37 ° C, 5 % CO_2_). One hour before cell imaging, 50 μl of medium was replaced with 50 μl of a cocktail of fluorescent probes containing: Hoechst 33342 (400 nM), Lysotracker Green (200 nM), TOPRO-3 (800 nM), and TMRM (20 nM). After 1 h incubation (37 ° C, 5 % CO_2_), cells were imaged using the Arrayscan VTI 740 (Thermo Scientific). During measurements standard cell conditions were maintained (37 ° C and 5 % CO_2_) through the Arrayscan Live Cell Module (Thermo Scientific). Plates were then read using 20× objective lens and fluorescent intensities were collected using four combinations of excitation/emission filters. Hoechst was visualized in the blue channel, Lysotracker in the green channel, TOPRO-3 and TMRM in the far-red and in the red, respectively. The utility of these dyes, their excitation/emission wavelengths and response profiling have been described previously [29]. For each well, ten independent fields were configured to image an average of 300 to 500 cells. Data were collected and analysed using the vHCS View software (Thermo Scientific) and the parameters investigated were: cell count (generated from the number of Hoechst stained nuclei), nuclear size (from the average object area of Hoechst), nuclear intensity, Lysotracker Green intensity and TOPRO-3 intensity. Analysis parameters were set according to manufacturer’s instructions and background noise was separated from fluorescence through fixed thresholds for each parameter. A specific algorithm was written in order to extract the quantitative data from images using the Cell Health Profile bioapplication.

### Hemolysis of human RBCs

RBCs were separated from fresh human blood of healthy volunteer donors not receiving any pharmacological treatment. The method for assessing the hemolytic activity induced by particles refers to [63], with minor modifications given in [53]. Hemolytic activity was evaluated exposing RBCs to increasing concentrations of quartz particles (from 6.25 to 200 cm^2^/ml) calculated on the basis of the BET surface area of each particle, as best metric for a surface-driven process. The haemoglobin released and measured spectrophotometrically at 540 nm did not adsorbed on quartz samples (data not shown).

### Lysis assay of artificial phosphatidylcholine unilamellar vesicles

#### Preparation and characterization

Calcein-loaded unilamellar vesicles were prepared by hydration of a dry lipid film and size exclusion chromatography. A dry film, obtained by the dissolution of 10 mg of phosphatidylcholine in a mixture of methanol-chloroform (1:2) followed by their removal with a rotary evaporator (Büchi Rotovapor R-200) and a stream of nitrogen gas, was hydrated with 4 ml of a 0.01 M calcein solution in 0.01 M PBS. The pH was adjusted to 7.4 with NaOH to ensure complete solubilisation of the calcein crystals. To separate the calcein-loaded vesicles from the free calcein molecules, a gel filtration step was performed. Approximately, 1 ml of liposome dispersion was loaded in a column filled with 10 ml Sepharose CL-4B beads, using 0.01 M PBS as eluent. Liposome dispersion was then diluted 1:8 with PBS and stored at +4 ° C. Liposome dispersion was characterized by dynamic light scattering (DLS) and **ζ** potential measurements (Zetasizer Nano–ZS, Malvern Instruments, Worcestershire, U.K.) (see Additional file 1: Figure S3). The **ζ** potential (at pH 7.4) was −7 ± 0.6 mV.

#### Leakage assay

Liposomes were incubated with increasing concentrations of quartz particles as for the hemolytic assay. Leakage of the fluorescent dye calceine (excitation and emission wavelength of 495 and 515 nm, respectively) encapsulated into liposomes was used as marker of liposome rupture. This method refers to [55, 68], with minor modifications. Briefly, liposomes dispersed in 0.01 M PBS were distributed in a 96-well plate (100 μL). A baseline fluorescence signal (intensity I_0_) was recorded with an excitation wavelength of 490 nm and an emission wavelength at 520 nm. Subsequently, particles dispersed in PBS were added to the final concentrations (in quadruplicate for each concentration) and fluorescence changes were monitored for 30 min (I_t_). Finally, a 20 % solution of Triton X-100 (10 μL) was added to completely disrupt the vesicles, and the fluorescence intensity (I_max_) of the complete release of the dye from the vesicles was determined. Percentage of the dye released from vesicles was calculated for each concentration with the following equation (Equation 1):

1 $$ \left[\left({\mathrm{I}}_{\mathrm{t}}\hbox{--}\ {\mathrm{I}}_0\right)\ /\ \left({\mathrm{I}}_{\max}\hbox{--}\ {\mathrm{I}}_0\right)\right] \cdot 100 $$

### ζ Potential

The ζ plot (ζ potential vs pH) for the four as-grown and fractured crystals was evaluated by means of electrophoretic light scattering (ELS) (Zetasizer Nano–ZS). In this technique the velocity of a particle in an oscillating electric field, which is proportional to its ζ potential, is measured by light scattering. The ζ potential was measured suspending quartz (0.6 mg/ml) in ultrapure water and adjusting the pH of the suspension with 0.1 M HCl or 0.1 M NaOH. A plot of ζ potential values versus pH was obtained. The set of experimental points was fitted with a sigmoid curve (Boltzmann equation) with OriginPro8.0 software suite (OriginLab Corp., Northampton, MA, USA), a simple approximation for the single-site equation model proposed by Sverjensky and Sahai [69] (Equation 2):

2 $$ y={A}_2+\frac{A_1-{A}_2}{1+{e}^{{\scriptscriptstyle \raisebox{1ex}{$\left(x-{x}_0\right)$}\!\left/ \!\raisebox{-1ex}{$dx$}\right.}}} $$

where *A*_*1*_ and *A*_*2*_ are the lower and upper horizontal asymptotes respectively, *x*_*0*_ the point of inflection and *dx* the curve rate, i.e. the change in *x* corresponding to the most significant change in *y* values.

### Solid state electron paramagnetic resonance (EPR) spectroscopy

The EPR spectra of the solid quartz dusts were recorded in vacuum at −196 ° C on a Bruker EMX spectrometer operating in the X–band mode (9.5 GHz), using a technique previously reported [33]. The spectra have been recorded with a scan range of 100 G (332–432 mT), receiver gain of 1x10^4^, microwave power of 0.1 mW, modulation amplitude of 1 G, and scan time of 80 s. Three scans per measurement were performed.

### Carbon-centred free radical detection

Free radical generation was monitored by EPR spectroscopy coupled with the spin trapping technique, using 5,5-dimethyl-pirroline-*N*-oxide (DMPO, Cayman chemical company, Ann Arbor, USA) as trapping agent, and following a well-established procedure [41, 70]. EPR spectra were recorded at room temperature on a Miniscope100 X– band CW- EPR spectrometer (Magnettech, Berlin, Germany) at a microwave power level of 10 mW, scan range of 120 G, and modulation amplitude of 1 G. Each quartz sample (37.5 mg) was suspended in 125 μL of 0.15 M DMPO. The reaction was triggered by adding formate ion (125 μL of 1.0 M HCOONa solution in 0.5 M PBS, pH 7.4) as a target molecule. The kinetic of radical release was progressively measured up to 1 h on an aliquot of 50 μL of the suspension. The amount of carbon-centred radicals generated is proportional to the intensity of the EPR signal after double integration. Each measurement was repeated three times.

### Statistical analysis

Statistical analysis was carried out by one-way analysis of variance (ANOVA) followed by Dunnett’s or Tukey’s *post hoc* tests, as appropriate. Differences with *p* value < 0.05 were considered statistically significant.

## Additional file

Additional file 1: Figure S1. Particle size distribution curves of the quartz crystals studied measured with DCS technique. **Figure S2.** Bio-TEM images of quartz samples internalized by RAW 264.7 murine macrophages. **Figure S3.** Size characterization curve of liposome dispersion measured by DLS. **Table S1.** Curve-fit parameters calculated by fitting experimental dataset (ζ potential vs pH) with a Boltzmann equation. (DOCX 952 kb)

## Abbreviations

BET: Brunauer, Emmett, and Teller method
DLS: dynamic light scattering
DMEM: Dulbecco’s modified Eagle medium
DMPO: 5,5-dimethyl-pirroline-*N*-oxide
DSC: differential centrifugal sedimentation
ECACC: European collection of authenticated cell cultures
EHT: extra high tension
ELS: electrophoretic light scattering
EPR: electron paramagnetic resonance
FBS: foetal bovine serum
FE-SEM: field emission scanning electron microscopy
HCA: high content analysis
HR-TEM: high resolution transmission electron microscopy
Na-MTS: sodium metasilicate pentahydrate
PBS: phosphate buffer solution
PS-NH_2_: amino-conjugated polystyrene beads
RBC: red blood cell
SD: standard deviation
SEM: scanning electron microscopy
SEM: standard error of the mean
SS-EPR: solid state EPR
WD: working distance

## References

[1] International Agency for Research on Cancer (IARC) (1997). Silica, Some Silicates, Coal Dust and Para-Aramid Fibrils. IARC Monographs on the Evaluation of Carcinogenic Risks to Humans.

[2] International Agency for Research on Cancer (IARC) (2012). A Review of Human Carcinogens: Arsenic, Metals, Fibres, and Dusts. IARC Monographs on the Evaluation of Carcinogenic Risks to Human.

[3] Ghiazza M, Polimeni M, Fenoglio I, Gazzano E, Ghigo D, Fubini B (2010). Does vitreous silica contradict the toxicity of the crystalline silica paradigm?. Chem Res Toxicol.

[4] Di Cristo L, Movia D, Bianchi MG, Allegri M, Mohamed BM, Bell AP, Moore C, Pinelli S, Rasmussen K, Riego-Sintes J (2015). Pro-inflammatory effects of pyrogenic and precipitated amorphous silica nanoparticles in innate immunity cells. Toxicol Sci.

[5] Zhang HY, Dunphy DR, Jiang XM, Meng H, Sun BB, Tarn D, Xue M, Wang X, Lin SJ, Ji ZX (2012). Processing pathway dependence of amorphous silica nanoparticle toxicity: colloidal vs pyrolytic. J Am Chem Soc.

[6] Gazzano E, Ghiazza M, Polimeni M, Bolis V, Fenoglio I, Attanasio A, Mazzucco G, Fubini B, Ghigo D (2012). Physicochemical determinants in the cellular responses to nanostructured amorphous silicas. Toxicol Sci.

[7] Sandberg WJ, Lag M, Holme JA, Friede B, Gualtieri M, Kruszewski M, Schwarze PE, Skuland T, Refsnes M (2012). Comparison of non-crystalline silica nanoparticles in IL-1 beta release from macrophages. Part Fibre Toxicol.

[8] Guidi P, Nigro M, Bernardeschi M, Scarcelli V, Lucchesi P, Onida B, Mortera R, Frenzilli G (2013). Genotoxicity of amorphous silica particles with different structure and dimension in human and murine cell lines. Mutagenesis.

[9] Napierska D, Thomassen LC, Rabolli V, Lison D, Gonzalez L, Kirsch-Volders M, Martens JA, Hoet PH (2009). Size-dependent cytotoxicity of monodisperse silica nanoparticles in human endothelial cells. Small.

[10] Merget R, Bauer T, Küpper HU, Philippou S, Bauer HD, Breitstadt R, Bruening T (2002). Health hazards due to the inhalation of amorphous silica. Arch Toxicol.

[11] Napierska D, Thomassen LCJ, Lison D, Martens JA, Hoet PH (2010). The nanosilica hazard: another variable entity. Part Fibre Toxicol.

[12] Maser E, Schulz M, Sauer UG, Wiemann M, Ma-Hock L, Wohlleben W, Hartwig A, Landsiedel R (2015). In vitro and in vivo genotoxicity investigations of differently sized amorphous SiO_2_ nanomaterials. Mutat Res Genet Toxicol Environ Mutagen.

[13] Guichard Y, Maire MA, Sébillaud S, Fontana C, Langlais C, Micillino JC, Darne C, Roszak J, Stępnik M, Fessard V (2015). Genotoxicity of synthetic amorphous silica nanoparticles in rats following short-term exposure. Part 2: intratracheal instillation and intravenous injection. Environ Mol Mutagen.

[14] Rabolli V, Badissi AA, Devosse R, Uwambayinema F, Yakoub Y, Palmai-Pallag M, Lebrun A, De Gussem V, Couillin I, Ryffel B (2014). The alarmin IL-1α is a master cytokine in acute lung inflammation induced by silica micro- and nanoparticles. Part Fibre Toxicol.

[15] Fubini B, Legrand AP (1998). Health effects of silica. The surface properties of silicas.

[16] Sankila R, Karjalainen S, Pukkala E, Oksanen H, Hakulinen T, Teppo L, Hakama M (1990). Cancer risk among glass factory workers: An excess of lung cancer?. Br J Ind Med.

[17] Guldner K (1999). Development of silicosis in the ceramics and glass industry. Gefahrst Reinhalt Luft.

[18] Fubini B (1998). Surface chemistry and quartz hazard. Ann Occup Hyg.

[19] Clouter A, Brown D, Hohr D, Borm P, Donaldson K (2001). Inflammatory effects of respirable quartz collected in workplaces versus standard DQ12 quartz: particle surface correlates. Toxicol Sci.

[20] Fubini B, Fenoglio I, Ceschino R, Ghiazza M, Martra G, Tomatis M, Borm P, Schins R, Bruch J (2004). Relationship between the state of the surface of four commercial quartz flours and their biological activity in vitro and in vivo. Int J Hyg Envir Health.

[21] Donaldson K, Stone V, Duffin R, Clouter A, Schins R, Borm P (2001). The quartz hazard: effects of surface and matrix on inflammogenic activity. J Environ Pathol Toxicol Oncol.

[22] Bruch J, Rehn S, Rehn B, Borm PJA, Fubini B (2004). Variation of biological responses to different respirable quartz flours determined by a vector model. Int J Hyg Environ Health.

[23] Stone V, Jones R, Rollo K, Duffin R, Donaldson K, Brown DM (2004). Effect of coal mine dust and clay extracts on the biological activity of the quartz surface. Toxicol Lett.

[24] Rimola A, Costa D, Sodupe M, Lambert JF, Ugliengo P (2013). Silica surface features and their role in the adsorption of biomolecules: computational modeling and experiments. Chem Rev.

[25] Donaldson K, Borm PJ (1998). The quartz hazard: a variable entity. Ann Occup Hyg.

[26] Pastero L, Turci F, Leinardi R, Pavan C, Monopoli M (2016). Synthesis of α-quartz with controlled properties for the investigation of the molecular determinants in silica toxicology. Cryst Growth Des.

[27] Murashov VV, Demchuk E (2005). A comparative study of unrelaxed surfaces on quartz and kaolinite, using the periodic density functional theory. J Phys Chem B.

[28] Margolis SVK, David H (1974). Processes of formation and environmental occurrence of microfeatures on detrital quartz grains. Am J Sci.

[29] Anguissola S, Garry D, Salvati A, O'Brien PJ, Dawson KA (2014). High content analysis provides mechanistic insights on the pathways of toxicity induced by amine-modified polystyrene nanoparticles. PLoS One.

[30] Jan E, Byrne SJ, Cuddihy M, Davies AM, Volkov Y, Gun'ko YK, Kotov NA (2008). High-content screening as a universal tool for fingerprinting of cytotoxicity of nanoparticles. ACS Nano.

[31] Iler RK (1979). The Surface Chemistry of Silica. The Chemistry of Silica: Solubility, Polymerization, Colloid and Surface Properties, and Biochemistry.

[32] Turci F, Ghibaudi E, Colonna M, Boscolo B, Fenoglio I, Fubini B (2010). An integrated approach to the study of the interaction between proteins and nanoparticles. Langmuir.

[33] Fubini B, Giamello E, Pugliese L, Volante M (1989). Mechanically induced defects in quartz and their impact on pathogenicity. Solid State Ionics.

[34] Ratdzig VA, Bystrikov AV (1978). ESR study of chemically active centers on the surface of quartz. Kinet Catal.

[35] Giamello E, Fubini B, Volante M, Costa D (1990). Surface oxygen radicals originating via redox reactions during the mechanical activation of crystalline SiO_2_ in hydrogen-peroxide. Colloid Surface.

[36] Fubini B, Giamello E, Volante M, Bolis V (1990). Chemical functionalities at the silica surface determining its reactivity when inhaled - formation and reactivity of surface radicals. Toxicol Ind Health.

[37] Vallyathan V, Shi XL, Dalal NS, Irr W, Castranova V (1988). Generation of free radicals from freshly fractured silica dust. Potential role in acute silica-induced lung injury. Am Rev Respir Dis.

[38] Castranova V, Dalal N, Vallyathan V, Castranova V, Vallyathan V, Wallace WE (1996). Role of surface free radicals in the pathogenicity of silica. Silica and silica-induced lung diseases.

[39] Fubini B, Hubbard A (2003). Reactive oxygen species (ROS) and reactive nitrogen species (RNS) generation by silica in inflammation and fibrosis. Free Radical Bio Med.

[40] Fubini B, Bolis V, Giamello E (1987). The surface chemistry of crushed quartz dust in relation to its pathogenicity. Inorg Chim Acta.

[41] Fenoglio I, Martra G, Prandi L, Tomatis M, Coluccia S, Fubini B (2000). The role of mechanochemistry in the pulmonary toxicity caused by particulate minerals. J Mater Synth Proces.

[42] Fenoglio I, Fonsato S, Fubini B (2003). Reaction of cysteine and glutathione (GSH) at the freshly fractured quartz surface: A possible role in silica-related diseases?. Free Radical Bio Med.

[43] Ghiazza M, Tomatis M, Doublier S, Grendene F, Gazzano E, Ghigo D, Fubini B (2013). Carbon in intimate contact with quartz reduces the biological activity of crystalline silica dusts. Chem Res Toxicol.

[44] Hemley RJ, Jephcoat AP, Mao HK, Ming LC, Manghnani MH (1988). Pressure-induced amorphization of crystalline silica. Nature.

[45] Li I, Bandara J, Shultz M (2004). Time evolution studies of the H_2_O/quartz interface using sum frequency generation, atomic force microscopy, and molecular dynamics. Langmuir.

[46] Jones RC, Uehara G (1973). Amorphous coatings on mineral surfaces. Soil Sci Soc Am J.

[47] Warheit DB, Webb TR, Colvin VL, Reed KL, Sayes CR (2007). Pulmonary bioassay studies with nanoscale and fine-quartz particles in rats: toxicity is not dependent upon particle size but on surface characteristics. Toxicol Sci.

[48] Øvrevik J, Refsnes M, Låg M, Holme JA, Schwarze PE (2015). Activation of proinflammatory responses in cells of the airway mucosa by particulate matter: oxidant- and non-oxidant-mediated triggering mechanisms. Biomolecules.

[49] Castranova V (2004). Signaling pathways controlling the production of inflammatory mediators in response to crystalline silica exposure: role of reactive oxygen/nitrogen species. Free Radic Biol Med.

[50] Albrecht C, Knaapen AM, Becker A, Höhr D, Haberzettl P, van Schooten FJ, Borm PJ, Schins RP (2005). The crucial role of particle surface reactivity in respirable quartz-induced reactive oxygen/nitrogen species formation and APE/Ref-1 induction in rat lung. Respir Res.

[51] Murashov V, Harper M, Demchuk E (2006). Impact of silanol surface density on the toxicity of silica aerosols measured by erythrocyte haemolysis. J Occup Environ Hyg.

[52] Fenoglio I, Fubini B, Ghibaudi EM, Turci F (2011). Multiple aspects of the interaction of biomacromolecules with inorganic surfaces. Adv Drug Deliv Rev.

[53] Pavan C, Tomatis M, Ghiazza M, Rabolli V, Bolis V, Lison D, Fubini B (2013). In search of the chemical basis of the hemolytic potential of silicas. Chem Res Toxicol.

[54] Pandurangi RS, Seehra MS, Razzaboni BL, Bolsaitis P (1990). Surface and bulk infrared modes of crystalline and amorphous silica particles: a study of the relation of surface structure to cytotoxicity of respirable silica. Environ Health Perspect.

[55] Alkhammash HI, Li N, Berthier R, de Planque MR (2015). Native silica nanoparticles are powerful membrane disruptors. Phys Chem Chem Phys.

[56] Nolan RP, Langer AM, Harington JS, Oster G, Selikoff IJ (1981). Quartz hemolysis as related to its surface functionalities. Environ Res.

[57] Prügger F, Mallner B, Schlipköter HW (1984). Polyvinylpyridine N-oxide (Bay 3504, P-204, PVNO) in the treatment of human silicosis. Wien Klin Wochenschr.

[58] Duffin R, Gilmour PS, Schins RPF, Clouter A, Guy K, Brown DM, MacNee W, Borm PJ, Donaldson K, Stone V (2001). Aluminium lactate treatment of DQ12 quartz inhibits its ability to cause inflammation, chemokine expression, and nuclear factor-kappa B activation. Toxicol Appl Pharm.

[59] Le Bouffant LDH, Martin JC (1977). The terapeutic action of aluminium compounds on the development of experimental lesions produced by pure quartz or mixed dust. Inhaled Part.

[60] Bégin R, Massé S, Sébastien P, Martel M, Bossé J, Dubois F, Geoffroy M, Labbé J (1987). Sustained efficacy of aluminum to reduce quartz toxicity in the lung. Exp Lung Res.

[61] Schins RP, Duffin R, Höhr D, Knaapen AM, Shi T, Weishaupt C, Stone V, Donaldson K, Borm PJ (2002). Surface modification of quartz inhibits toxicity, particle uptake, and oxidative DNA damage in human lung epithelial cells. Chem Res Toxicol.

[62] Peeters PM, Eurlings IMJ, Perkins TN, Wouters EF, Schins RPF, Borm PJA, Drommer W, Reynaert NL, Albrecht C (2014). Silica-induced NLRP3 inflammasome activation in vitro and in rat lungs. Part Fibre Toxicol.

[63] Lu SL, Duffin R, Poland C, Daly P, Murphy F, Drost E, MacNee W, Stone V, Donaldson K (2009). Efficacy of simple short-term in vitro assays for predicting the potential of metal oxide nanoparticles to cause pulmonary inflammation. Environ Health Persp.

[64] Cho WS, Duffin R, Bradley M, Megson IL, MacNee W, Lee JK, Jeong J, Donaldson K (2013). Predictive value of in vitro assays depends on the mechanism of toxicity of metal oxide nanoparticles. Part Fibre Toxicol.

[65] Pavan C, Rabolli V, Tomatis M, Fubini B, Lison D (2014). Why does the hemolytic activity of silica predict its pro-inflammatory activity?. Part Fibre Toxicol.

[66] Monopoli MP, Walczyk D, Campbell A, Elia G, Lynch I, Bombelli FB, Dawson KA (2011). Physical-chemical aspects of protein corona: relevance to in vitro and in vivo biological impacts of nanoparticles. J Am Chem Soc.

[67] George S, Pokhrel S, Xia T, Gilbert B, Ji Z, Schowalter M, Rosenauer A, Damoiseaux R, Bradley KA, Mädler L, Nel AE (2010). Use of a rapid cytotoxicity screening approach to engineer a safer zinc oxide nanoparticle through iron doping. ACS Nano.

[68] Ladokhin AS, Wimley WC, White SH (1995). Leakage of membrane vesicle contents: determination of mechanism using fluorescence requenching. Biophys J.

[69] Sverjensky DA, Sahai N (1996). Theoretical prediction of single-site surface-protonation equilibrium constants for oxides and silicates in water. Geochim Cosmochim Acta.

[70] Fubini B, Mollo L, Giamello E (1995). Free-radical generation at the solid/liquid interface in iron-containing minerals. Free Radical Res.

