# Supplementary material for: Revisiting the paradigm of silica pathogenicity with synthetic quartz crystals: the role of crystallinity and surface disorder
Source: Part Fibre Toxicol. 2016 Jun 10;13:32. doi: 10.1186/s12989-016-0136-6 (PMC4902968; doi:10.1186/s12989-016-0136-6)
Supplement: Additional file 1: Figure S1. — Particle size distribution curves of the quartz crystals studied measured with DCS technique. Figure S2. Bio-TEM images of quartz samples internalized by RAW 264.7 murine macrophages. Figure S3. Size characterization curve of liposome dispersion measured by DLS. Table S1. Curve-fit parameters calculated by fitting experimental dataset (ζ potential vs pH) with a Boltzmann equation. (DOCX 952 kb) [file 12989_2016_136_MOESM1_ESM.docx]

Additional file 1

The pathogenicity of silica, crystallinity and surface disorder: revisiting the paradigm with synthetic quartz crystals

*Francesco Turci, Cristina Pavan, Riccardo Leinardi, Maura Tomatis, Linda Pastero, David Garry, Sergio Anguissola, Dominique Lison, Bice Fubini*

**Fig. S1** Particle size cumulative distribution curves measured by DCS technique. The particle size of the two as-grown crystals was largely different, being the average particle diameter of n-Qz-syn << μ-Qz-syn (50^th^ percentile of the cumulative size distribution at ca. 280 nm and 900 nm, respectively). Particle size distribution of fractured quartz crystals (μ-Qz-syn-f) was similar to mineral quartz dust (Qz-f).


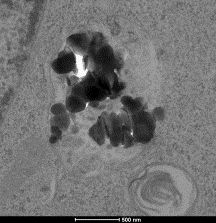

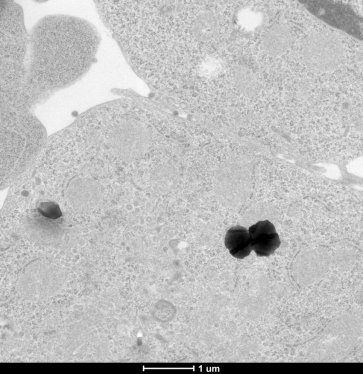

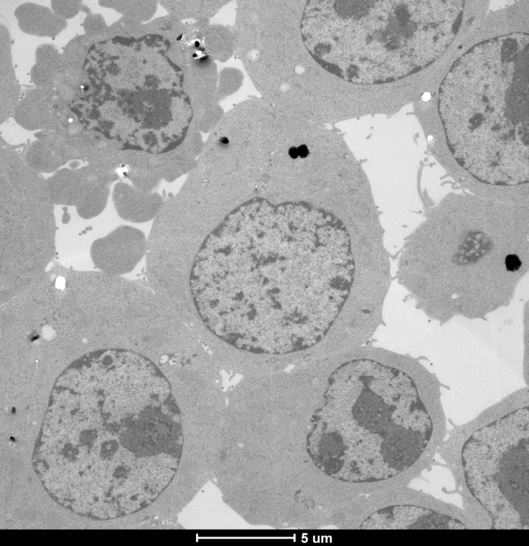

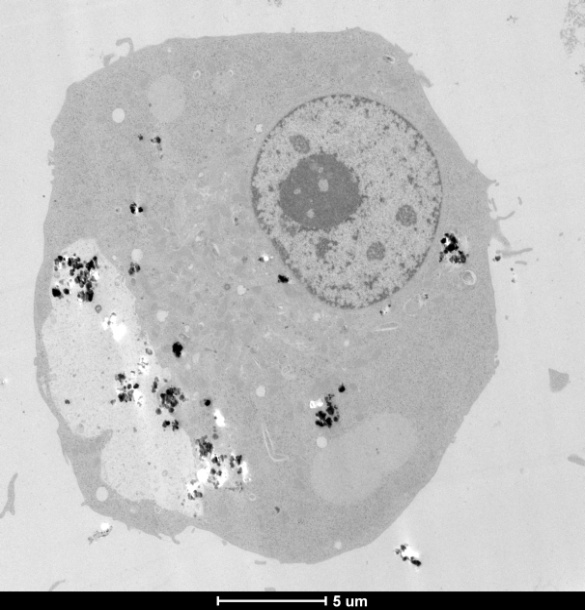


**B**

**A**


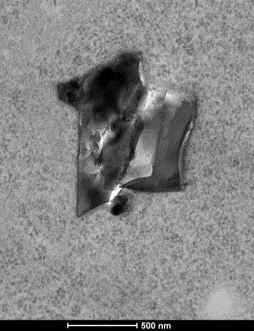

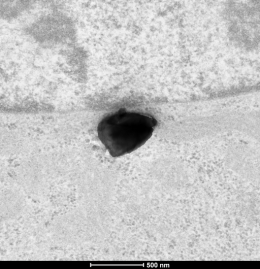

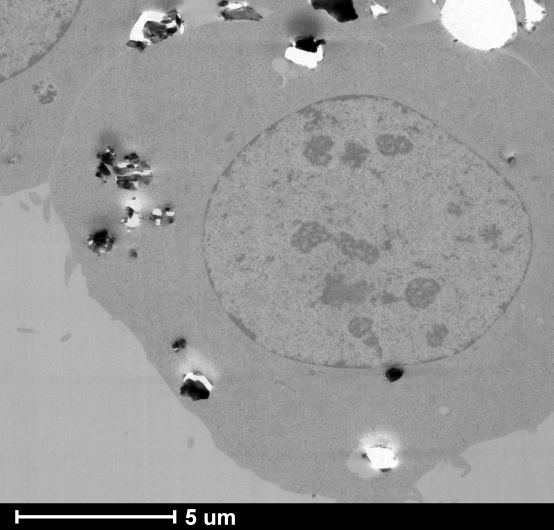

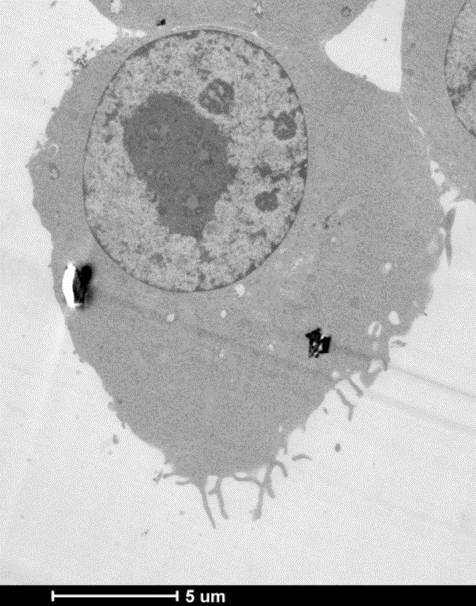


**D**

**C**

**Fig. S2** Bio-TEM images of quartz samples internalized by Raw264.7 murine macrophage cell line. Cells were exposed to 100 µg/ml of as-grown (n-Qz-syn and μ-Qz-syn) and fractured (μ-Qz-syn-f and Qz-f) quartz (A, B, C, and D, respectively) for 24h and thin cross-sections of fixed cell observed at low and high (insets) magnification. Internalization was observed at all exposures investigated, with particles (aggregates and largest particles) mainly localized in lysosomal compartments. In some cases, the fusion of lysosomes in larger compartments could be observed (panel B), but without any sign of nuclear uptake of intact particles. Scale bars: 5 μm, black and 500 nm, white.TEM imaging was performed accordingly to [1].

**Fig. S3** Size characterization curve of liposome dispersion measured by DLS, expressed in intensity (%) *vs* size (nm). After being synthesized, liposomes were dispersed in 0.01 M phosphate-buffered saline (PBS, pH 7.4), and characterized for their hydrodynamic size through dynamic light scattering technique (DLS). The first peak (at ca. 50 nm) is representative of small unilamellar vesicles (SUVs), usually characterized by a diameter of 20 nm up to 100 nm. The second peak (at ca. 280 nm) is representative of large unilamellar vesicles (LUVs), usually ranging from 100 nm up to few micrometers. In our dispersion, LUVs ranges from 120 to 580 nm.

**Table S1** Curve-fit parameters calculated by fitting experimental dataset (ζ potential *vs* pH) with a Boltzmann equation

|  | | n-Qz-syn | μ-Qz-syn | μ-Qz-syn-f | Qz-f |
| --- | --- | --- | --- | --- | --- |
| *Ave*. pK_a_^§^ | 3.08 ± 0.07 | | 3.21 ± 0.11 | 2.77 ± 0.11 | 4.65 ± 0.06 |
| *f* '(pK_a_)^†^ | -32.9 | | -23.2 | -18.6 | -14.4 |

^§^ the point of inflection

^†^ the curve slope at the point of inflection

When quartz is suspended in water an equilibrium is readily established between protonated and dissociated silanols [2]:

-SiO^−^ + H_2_O ⮀ -SiOH + OH^−^

In an alkaline environment the vast majority of silanols will be dissociated with a net surface charge markedly negative (ca. -65 mV). As the pH decreases, the protonated form is favoured and the overall surface charge becomes less negative. At pH < 2, all silanols are in the protonated form and the net charge of quartz is virtually zero. This pH value corresponds to the PZC, which in quartz can reasonably considered asymptotic, since the formation of surface species SiOH_2_^+^ is not expected for silica [3].

As reported in the Methods section, the experimental ζ potential points can be fitted with a non-linear Boltzmann equation. Two parameters can be readily extrapolated, namely the point of inflection (x_0_) and the curve slope at the point of inflection (*f* ' (x_0_)), i.e. the change in x corresponding to the most significant change in y values. Those empirical parameters may conveniently be used to describe the “average acidity” of surface silanols (*Ave*. pK_a_ = x_0_) and the intrinsic variability of acidic character showed by the families of silanols (curve slope at the point of inflection, (*f* ' (pK_a_)).

The steeper slope (*f* ' (pK_a_)) observed for n-Qz-syn and μ-Qz-syn may account for a less heterogeneous distribution of surface acidic sites (i.e. silanols) with respect to fractured quartz (μ-Qz-syn-f and Qz-f). We may infer that the slope decreases by increasing the heterogeneity of the surface acidic silanols as it occurs after fragmentation of the crystals during milling. While as-grown quartz crystals expose intact crystal planes (n-Qz-syn and μ-Qz-syn), fractured quartz (Qz-syn-f and Qz-f) is characterized by conchoidal fractures, imparting a more heterogeneous silanol composition on quartz surface. Therefore, depending on the heterogeneity of the surface acidic silanols, the curve slope of the tangent line may vary, and it may represent an indirect measure of the surface chemical homogeneity of a quartz particle.

**References**

1. Lesniak A, Fenaroli F, Monopoli MP, Åberg C, Dawson KA, Salvati A: **Effects of the presence or absence of a protein corona on silica nanoparticle uptake and impact on cells.** *ACS Nano* 2012, **6:**5845-5857.

2. Iler RK: **The Surface Chemistry of Silica.** In *The Chemistry of Silica: Solubility, Polymerization, Colloid and Surface Properties, and Biochemistry.* Edited by Wiley. New York; 1979:622-729.

3. Liu X, Cheng J, Lu X, Wang R: **Surface acidity of quartz: understanding the crystallographic control.** *Phys Chem Chem Phys* 2014, **16:**26909-26916.
